# School for the Blind

**Published:** 1843-01

**Authors:** 


### SCHOOL FOR THE BLIND.

It affords us great satisfaction to be able to announce, that the State, in connexion with our city, has established the school for the blind, of which we have already made mention more than once. It is now in actual operation, and cannot fail to grow, and prove a great blessing to a class of sufferers dear to the heart of every medical man; because their infirmity is the offspring of diseases of the eye, or of our mistakes in the treatment of them. Whether from one cause or the other, the blind are our beneficiaries, and we should labor for their education and comfort. In every county of the State, there are blind children and youth of both sexes, whose parents are ignorant of their capabilities, under appropriate means of instruction, and equally unacquainted with the character of the latter, so honorable to the inventive genius and benevolence of our age. It is the duty, as it should be the happiness, of physicians, to seek out such families, and enlighten them in regard to a school, which ought to embrace all the uneducated blind of the State, and train them up to letters, music, and useful handicraft occupations. Letters requesting information concerning the school, may be addressed to Mr. Bryce M. Patten, who will promptly and cheerfully give all the information required. D.

